# Implementation of an Oncogeriatric Unit for Frail Older Patients with Breast Cancer: Preliminary Results

**DOI:** 10.3390/curroncol31120575

**Published:** 2024-12-04

**Authors:** Helena Hipólito-Reis, Joana Santos, Paulo Almeida, Luciana Teixeira, Fernando Rodrigues, Nuno Teixeira Tavares, Darlene Rodrigues, Jorge Almeida, Fernando Osório

**Affiliations:** 1 Department of Internal Medicine, São João University Hospital (ULS São João), 4200-319 Porto, Portugal; joana.gouveia.santos@ulssjoao.min-saude.pt (J.S.); paulo.sousa.almeida@ulssjoao.min-saude.pt (P.A.); jorge.salmeida@ulssjoao.min-saude.pt (J.A.); 2 Department of Nutrition, São João University Hospital (ULS São João), 4200-319 Porto, Portugal; luciana.lima.teixeira@ulssjoao.min-saude.pt; 3 Hospital Epidemiology Center, São João University Hospital (ULS São João), 4200-319 Porto, Portugal; fernando.rodrigues@ulssjoao.min-saude.pt; 4 Department of Oncology, São João University Hospital (ULS São João), 4200-319 Porto, Portugal; nuno.tavares@ulssjoao.min-saude.pt; 5 Breast Center, São João University Hospital (ULS São João), 4200-319 Porto, Portugal; 6 Department of Behavioural Sciences, Institute of Biomedical Sciences Abel Salazar, 4050-313 Porto, Portugal; darlenerodrigues.dr@outlook.pt; 7 RISE@CINTESIS-Center for Health Technology and Services Research, 4200-450 Porto, Portugal; 8 Department of Medicine, Faculty of Medicine, University of Porto, 4099-002 Porto, Portugal; 9 Department of Surgery and Physiology, Faculty of Medicine, University of Porto, 4099-002 Porto, Portugal

**Keywords:** breast neoplasms, frailty, geriatrics, ageism, geriatric assessment, undertreatment

## Abstract

(1) Background: Breast cancer (BC) has a high incidence in Europe, particularly in older adults. Traditionally under-represented in clinical trials, this age group is often undertreated due to ageism. This study aims to characterize frail older adults (≥70 years) with BC based on a comprehensive geriatric assessment, to guide individualized treatment decision-making. (2) Methods: A descriptive analysis of older adults with BC treated from January 2021 to December 2022 was performed. Data were analyzed based on anonymized electronic medical records. (3) Results: Of 123 patients (mean age 84.0 ± 5.6 years), 122 (99.2%) were women. The mean G8 screening score was 12.1 ± 2.5. Most had functional dependence (69.9% Barthel Index, 81.3% Lawton/Brody Scale) and a moderate-to-high risk of falling (76.4% Tinetti index). Cognitive impairment and malnutrition risk were present in 15.4% and 30.1%, respectively. Prehabilitation inclusive strategies led to adapted treatment in 55.3% of cases. Endocrine therapy, surgery, radiotherapy, and chemotherapy was used in 99.2%, 56.1%, 35.0%, and 8.9% of patients, respectively. (4) Conclusions: Our comprehensive oncogeriatric strategy promotes personalized oncologic treatment, improves outcomes by addressing frailty, and enhances treatment tolerability in older patients with BC, validating the expansion of this combined team approach to other cancer types and institutions.

## 1. Introduction

As the world’s population ages, the incidence and prevalence of cancer is growing in older adults, who represent 70% of cancer patients [1]. According to the World Health Organization (WHO), cancer was the leading cause of death worldwide in 2020 [2]. In Europe, breast cancer (BC) is the most common malignancy among women, particularly in older women, making it one of the leading causes of death in women over 65 years [2]. In Portugal, according to data from the National Cancer Registry, there were 7504 new BC diagnoses in 2020, of which, 2270 (30.3%) were in patients over 70 years [3]. In the same year, according to the WHO, BC was the leading cause of cancer-related death among Portuguese women, responsible for 1864 deaths, of which, 1103 were in patients over 70 years [2]. With increasing life expectancy, BC has become a public health priority as a significant yet possibly preventable cause of mortality in older women. 

However, despite the high prevalence of cancer, older adults are under-represented in clinical trials, leading to limited specific cancer treatment recommendations [4]. The physiological changes in the elderly, such as decreased lean mass and increased fat mass which affect the pharmacokinetic drugs, have a significant impact on cancer treatment [5]. The inter- and intra-individual heterogeneity in older people leads to a gap in knowledge about how these clinical particularities, as well as geriatric syndromes (GSs), interact with multimodal cancer treatment [6]. As a result, ageism (prejudice based on age) by patients and the multidisciplinary team (MDT) is often responsible for the not infrequent undertreatment of older adults with cancer [7,8]. It is crucial to adapt current oncologic recommendations to older patients with cancer, considering their physiologic heterogeneity, comorbidities, patient and family preferences, life expectancy, therapeutic iatrogeny and evidence of benefit for each proposed treatment to minimize undertreatment and overtreatment [1]. Furthermore, few studies contemplate the impact of cancer and its treatment on quality of life (QoL), a condition highly valued by older adults [6]. Many of them prioritize functionality and QoL over longevity [9].

The European Society of Breast Cancer Specialists (EUSOMA) and the International Society of Geriatric Oncology (SIOG) updated their recommendations for BC management in older adults in 2021 [10]. A frailty screening in patients over 70 years was advocated for, with cancer treatment adjusted based on patient categorization (fit, vulnerable/pre-frail or frail). Mortality and the recurrence risk of the cancer, as well as the competitive risk of death from other causes, should also be considered, as comorbidities have a greater impact on elderly than on younger adults. Toxicity scales (e.g., CARG—Cancer and Aging Research Group Chemotherapy Toxicity Tool, or CRASH—Chemotherapy Risk Assessment Scale for High-age) can be used as well to estimate chemotherapy iatrogenic risk [10]. These recommendations emphasize that the functional status, comorbidities, and life expectancy of each patient are key factors in the decision-making, highlighting the importance of comprehensive geriatric assessment (CGA). Traditionally, surgery remains the standard of care for most older adults [10]. It has a proven survival benefit with no negative impact on QoL in fit patients [11,12]. Given the inevitably lower risk of recurrence, the real benefit of adjuvant radiotherapy after conservative breast surgery in older adults is more debatable [10]. Primary endocrine therapy is an effective and well-tolerated option for frail patients with luminal BC [10]. Chemotherapy and targeted therapies, such as anti-HER2 blockade or immunotherapy, may be considered based on both patient and cancer characteristics [10]. As stated, a risk–benefit assessment, as well as potential adverse effects, must be considered [10].

Chronological age should not be the only criteria for defining oncologic treatment, as recommended by several international scientific societies (SIOG; National Comprehensive Cancer Network—NCCN; European Organization for Research and Treatment of Cancer—EORTC; American Society of Clinical Oncology—ASCO), which advocate the use of CGA in older patients with cancer [6,9]. The recent ASCO and SIOG position paper reaffirms that CGA should be conducted in older adults over 70 years and, if possible, in those over 65 years when cancer treatments, especially systemic therapies, are being considered, and ideally as early as possible (before treatment initiation) [13]. Although CGA should ideally be performed in all older adults with cancer, regardless of frailty, resource limitations often limit its use to frail patients [14]. The ASCO/SIOG Cancer in the Elderly Working Group proposes different CGA implementation models based on local resources and expertise [13]. The more traditional model, considered the gold standard, involves MDT (including a geriatrician, oncologist, nutritionist, social worker, and occupational/rehabilitation therapist), ideally with the CGA performed in one place and at one time to minimize hospital visits for older adults [13]. Due to the time and specialized resources required for this model, other alternative co-management models are possible: the share–care model, in which the MDT performs the evaluation at different times and the therapeutic plan is developed later; and the two-step consultative model, in which a screening scale is used to identify at-risk patients who would benefit from CGA by a geriatrician, whose recommendations are then provided to the oncologist [13].

CGA is a multidimensional and interdisciplinary assessment that is essential to accurately and comprehensively identify all health problems in older adults, especially comorbidities and GSs. Simultaneously, it allows for the detailed assessment of specific domains, such as functional, cognitive, nutritional or social status. This can improve prognosis and life expectancy in older adults [1,9,13]. Moreover, CGA is not solely a diagnostic tool that addresses both oncologic and non-oncologic needs. It also incorporates multidimensional prehabilitation strategies to improve patient outcomes and to individually tailor and support the management and tolerance of cancer treatment [9,13,14]. A systematic review of 61 studies found that non-oncological interventions were recommended for approximately 70% of patients who underwent CGA, with a primary focus on social support, nutrition and polypharmacy [9]. 

Our objective was to characterize the model of CGA that we implement in clinical practice, at the Oncogeriatric Unit of the São João University Hospital. Our oncogeriatric strategy aims to achieve the multidimensional characterization, prehabilitation and support of older adults with BC to promote personalized oncologic treatment. In addition, we aimed to describe the prehabilitation strategies of our interdisciplinary team to improve outcomes and treatment tolerance in cancer patients.

## 2. Materials and Methods

We conducted a retrospective, non-interventional cohort study of patients followed at the Oncogeriatric Unit of São João University Hospital, Porto, Portugal, over a two-year period (from January 2021 to December 2022). 

Our referral protocol included an initial frailty screening by the referring physician who applied the G8 scale to patients aged 70 years or more with a new BC diagnosis. Patients scoring ≤14 (indicating frailty) [15] were referred to the multidisciplinary oncogeriatric team—including an internist with geriatric expertise, a rehabilitation nurse, a social worker, a psychologist, and a nutritionist—who performed a CGA. A sequential holistic evaluation included the assessment of functional status using the Barthel index [16] (for basic daily activities essential for independent living, with higher scores indicating better performance), the Lawton/Brody scale [17] (for instrumental daily activities needed for community living, with higher scores indicating better performance) and ECOG-PS (Eastern Cooperative Oncology Group—Performance Status) [18], with lower scores indicating better performance. The rehabilitation nurse then assessed the performance status, gait and balance using the functional ambulation category [19] and Tinetti index [20], with higher scores indicating better performance, and the time up and go test [21] and five times sit-to-stand test [22], with longer times indicating worse performance. The number of falls in the past six months was reviewed [9,13]. The cognitive status was screened initially using open questions to identify any memory complaints, disorientation or cognitive impairment. If any concerns were noted, further assessments were conducted, including mini mental state examinations (MMSEs) [23], with higher scores indicating better cognitive status, and the clock drawing test [24]. This domain also included screening for depression risk using the geriatric depression scale [25], with lower scores indicating lower risk. Nutritional status was assessed through anthropometric measurements (recent weight loss, body mass index [BMI]) [9,13] as well as mini nutritional assessment (MNA) [26], with lower scores indicating higher risk. Social status was evaluated through questions on social support, living arrangements, housing conditions and architectural barriers. Additionally, the team reviewed each patient’s comorbidities, medical history, medication and immunizations and calculated mortality and prognosis estimates [13]. To identify GSs [13], patients or caregivers were asked about polypharmacy, visual and hearing impairment, incontinence, constipation, swallowing difficulties and pressure ulcers. Finally, several interventions were proposed in each domain based on the identified issues, along with prescribed prehabilitation strategies. These included the optimization of comorbidities and GSs, therapeutic reconciliation, immunization updates, fall prevention, functional and motor rehabilitation through Otago exercises [27], protein reinforcement or other nutritional reinforcement, social support guidance and cognitive therapy or emotional support. When necessary, patients were also referred for further evaluation by specialists in a more targeted consultation. Following this assessment pathway, each patient was discussed in an oncogeriatric MDT to define the geriatric perspective and support needs, and then discussed in the oncology MDT to select and individualize the most appropriate cancer treatment.

An anonymized qualitative and quantitative description of the oncogeriatric assessment pathway was provided. Descriptive statistics were performed using IBM SPSS Statistics 29.0^®^. The Ethics Committee of São João University Hospital approved this study (302/22, 2022).

## 3. Results

In total, 123 older adults were studied; 99.2% (*n* = 122) were women. The mean age was 84.0 (SD ± 5.6). The mean G8 score was 12.1 (SD ± 2.5). Initially administered as a screening tool by the referring physician, this scale was reassessed during the oncogeriatric consultation and occasionally showed some variability due to interobserver differences. The reported values represent the second assessment.

Table 1 outlines the multidimensional assessment conducted by our interdisciplinary team. Regarding functional status, 70% of patients showed moderate-to-total dependence (≤95 points) in basic daily living activities according to the Barthel index [16], and 81% had mild-to-total dependence (men ≤ 4 and women ≤ 7) in instrumental daily living activities per the Lawton/Brody scale [17]. However, based on the ECOG-PS [18] score, 68% of patients had scores between 0 and 2. Additionally, 76% of patients exhibited moderate-to-high risk of falls (≤23 points) according to the Tinetti index (gait and balance evaluation) [20]. In terms of cognitive status, 15% of patients demonstrated cognitive impairment according to the MMSE screening [23]. Approximately 46% of patients were not evaluated for this aspect, as they did not report memory complaints and thus did not require further cognitive assessment. Regarding nutritional status, 15% of the population had a BMI [28] below 22 (underweight). However, the MNA [26] revealed that 30% of patients were at risk of malnutrition (8–11 points). In our cohort, 59% of patients presented with four or more GSs, with the most prevalent being polypharmacy (83%), visual impairment (70%), urinary incontinence (55%) and hearing loss (49%)—Table 2.

The most frequently implemented prehabilitation strategies included fall prevention (100%), updating immunizations (100%), providing social support guidance (100%), therapeutic reconciliation (98%), comorbidity optimization and stratification (93%), functional and motor rehabilitation (83%) using Otago exercises, protein reinforcement/nutritional reinforcement/oral nutritional supplements (40%) and cognitive therapy/emotional support (30%).

The interaction of oncogeriatric and oncologic MDT allowed for personalized treatment. Multimodal treatment included endocrine therapy (99%), surgery (56%), radiotherapy (35%) and chemotherapy (9%). Standard cancer treatment according to national and international guidelines was possible in 21% of our frail patients. In 55% of our cohort, tailored and adapted treatment was proposed. Symptomatic and palliative care was offered to 24% of patients. 

## 4. Discussion

Internationally recognized as the gold standard, CGA is defined as a holistic, interdisciplinary, and multidimensional diagnostic process to assess functional capacity, physical and mental health, social and family circumstances and spirituality to identify needs, plan individualized care and improve health outcomes and survival in older patients [6,9,13,29,30]. CGA integrates multiple dimensions, perspectives and parameters—clinical and non-clinical. There are seven essential ones, cognitive, functional, nutritional, social, mobility and falls, multimorbidity and polypharmacy, and each must be assessed using specific, validated and standardized measurement tools. CGAs are time-consuming, demanding and somewhat complex, as they are aimed at older adults with multiple and complex needs. In the absence of a single, standardized CGA model, there is a clear consensus in the literature about its utility and how to implement it in clinical practice [14,31,32]. The scarcity of comparative studies for this multidisciplinary assessment challenges the standardization of tools and models of care. A study shows CGA influences the treatment plan in 25% of older adults with BC [33]. Another study showed that 40% of oncologists’ initial treatment plans were modified based on CGA [34].

The traditional geriatric classification of fit, vulnerable, and frail patients is helpful and convenient in oncology. Fit older adults are usually candidates for standard cancer treatment. Vulnerable and frail patients derive the greatest clinical benefit from CGA, highlighting the importance of identifying them [35]. We selected the G8 tool for initial screening for frailty because it is quick and intuitive to use, has been validated in cancer patients, has high sensitivity, has negative predictive value and prognostic value and is an independent predictor of mortality risk [15,36,37,38]. However, screening for frailty is not enough in oncogeriatrics. In older adults screened as frail, it is mandatory to perform a CGA with a diagnostic and interventional purpose.

Regarding physical performance, the ECOG-PS scale is the most widely used functional assessment tool in oncology [9,39]. However, this scale tends to underestimate functional dependence in older adults when compared to basic and instrumental activities of daily living (ADL) scales [9,39,40]. A study of 105 patients over 70 years old followed in an oncogeriatric unit showed that 42% of patients required assistance with basic ADLs and 54% with instrumental ADLs, although only 7% had an ECOG-PS score >2 [41]. Our cohort also illustrates a clear discrepancy between ECOG-PS scores and dependence in both basic and instrumental ADLs. Therefore, we can infer that the ECOG-PS scale alone is not an adequate tool for assessing the physical performance of older adults. Thus, other functional assessment scales, such as the Barthel Index or Lawton/Brody scale used in this study, should be considered to more accurately evaluate the functional capacity of older adults. Other scales used to assess physical performance, such as the Tinetti index, the TUGT and the five times sit-to-stand test, should also be applied to strengthen the robustness of the assessment.

Cognitive decline has a significant impact on life expectancy and adherence to cancer treatment [40], making cognitive screening in older adults crucial. After screening for cognition impairment, patients with suspected dementia are referred for neuropsychological evaluation or directed to specialized consultations. Depression is another frequent clinical condition among older adults. However, the Geriatric Depression Scale (Yeasavage) [25] used in our protocol has limitations, particularly when caregivers are present during consultations, as we have observed in clinical practice, patients feel uncomfortable expressing their concerns. Despite this, our team is attuned to these challenges and actively works to address and manage the presence of depression.

Regarding nutritional status, the BMI is a simple and widely used tool in oncology, but it may not correlate with malnutrition risk, making it inadequate for nutritional assessment in older adults. Additionally, there are specific BMI cutoffs for the geriatric population due to physiological changes related to aging (such as reduced height, increased fat mass, and decreased muscle mass). However, these adaptations are not well known or frequently used, leading to the overestimation of nutritional status in older adults [42]. Our cohort, even by using BMI adapted for older adults, illustrates that BMI alone is insufficient for assessing the nutritional status. It is essential to use malnutrition screening tools that offer a more precise and appropriate evaluation, such as the MNA [43]. Using the short form of this tool, malnutrition or malnutrition risk was identified in 35% of patients—over twice the rate identified as underweight by BMI. This finding underscores the importance of using additional assessment tools beyond BMI to more accurately evaluate older adults and identify those at high risk who would benefit from prehabilitation by the team’s nutritionist with individualized caloric-protein reinforcement strategies.

Our study highlights the importance of diagnosing and intervening in GSs, as nearly 60% of patients presented with four or more of these syndromes. Falls and polypharmacy are common in older patients, especially those with cancer, and can increase the risk of adverse effects [44]. Falls are often linked to sarcopenia and malnutrition—conditions common among older adults and associated with poor health outcomes. Most patients in our study exhibited a moderate-to-high risk of falling, as assessed by the Tinetti index, as well as polypharmacy. The GAP70+ study showed that patients not assessed by a CGA had a higher prevalence of falls, with over 20% experiencing a fall within 3 months of starting a new treatment [44]. 

Our oncogeriatric strategy reinforces the value of implementing a CGA in clinical practice to manage older adults with cancer. Most older adults evaluated in our clinic had functional decline (some degree of functional dependence), had a moderate-to-high risk of falls, and were at risk for or already experiencing malnutrition. In addition, some had cognitive impairment and a significant percentage had at least four GSs. All these factors increase the likelihood of complications during multimodal cancer treatment, inevitably leading to adaptation and tailoring adjustments or even the discontinuation, namely through the decision not to treat, of appropriate treatments, as well as further deterioration of these patients’ baseline statuses. 

Prehabilitation, as a component of CGA, thus becomes a key intervention in older adults, allowing for the optimization of clinical status through dynamic strategies that correct deficits in multiple geriatric domains and improve comorbidities [33]. The INTEGRATE study showed that oncogeriatric care based on CGA reduced the impact of treatment on functional status, decreasing discontinuation due to toxicity [33]. Similarly, the GERICO study found that CGA performed by a geriatrician helped frailer patients complete treatment with a lower disease burden and morbidity [45]. Therapeutic reconciliation and deprescription is another key intervention in our strategy due to the high prevalence of polypharmacy in older adults [40]. This population typically uses four to nine medications [40], with a study of 105 BC patients over 70 revealing that 74% took three or more medications [41]. In our study, 83% of patients were on five or more drugs. CGA played a critical role in reducing polypharmacy, minimizing drug interactions and side effects during cancer treatment [40].

Interdisciplinary teamwork, motivation, resilience and awareness are essential skills for working in oncogeriatrics. Managing cancer in older adults, as in younger age groups, as a preventable cause of death is challenging and requires the simultaneous consideration of survival and QoL. Adapted and tailored multimodal cancer treatment is usually warranted in frail older adults. The interaction and co-management of oncogeriatric MDT and oncologic MDT is critical to this therapeutic individualization, to respect patients’ preferences and to minimize both the undertreatment and overtreatment [6,9,13,29]. The GAP70+ study emphasized the importance of sharing CGA results with the oncology care team, showing that this approach helped in decision-making and treatment personalization [44]. The GAIN study reinforced that interventions for older patients with cancer should be based on CGA and discussed by an MDT trained in geriatrics [46]. In our study, all frail patients were discussed in an oncogeriatric MDT, and most of them underwent multidimensional prehabilitation, and subsequently were discussed in the oncology MDT to define an individualized treatment plan: the majority (55%) received tailored, non-conventional BC treatment (for example, primary endocrine therapy only or conservative breast surgery without adjuvant radiotherapy); standard BC treatment according to national and international guidelines as for younger patients was possible in 21% of our frail older adults; symptomatic and palliative care was offered to 24% of patients. In the future, this cohort of patients will be followed to evaluate the impact of the proposed prehabilitation on the tolerability of multimodal cancer treatment, functional outcomes, overall survival and BC-specific survival and QoL.

Our study has some limitations. Firstly, it only includes patients with BC, the vast majority of whom were women. This makes it less representative, although several studies demonstrate the clear benefit of CGA in different types of cancers. Another limitation of its representativeness was the inclusion of only frail and vulnerable patients without distinguishing between these groups. The third limitation was the lack of the systematic assessment of QoL, despite being one of the most important domains reported by older adults and a topic frequently highlighted in the literature. We plan to include this assessment in future studies. A challenge, more than a limitation, we encountered was the scarcity of human resources dedicated to geriatrics, which limits the replication of this approach for other types of cancers. 

## 5. Conclusions

A systematized and standardized CGA is the needed clinical pathway to personalize oncologic treatment for older adults, especially those who are frail. It allows for a thorough and accurate diagnosis of age-related issues and limitations that may affect cancer treatment. With an emphasis on functionality and QoL, CGA should include prehabilitation measures to optimize clinical status, reduce or even reverse frailty and promote more effective therapeutic options. Combined oncogeriatric diagnostics and prehabilitation is critical to shifting the paradigm in the decision-making co-management of older patients with cancer. 

Our study illustrates the preliminary results of a pioneering oncogeriatric unit in Portugal. Demonstrating the implementation and outcomes of our strategy for frail older adults with BC, we intend to extend its applicability to other types of cancer in our hospital and promote its dissemination to more hospitals to improve a patient-centered approach to older adults with cancer. 

## Figures and Tables

**Table 1:** Comprehensive geriatric assessment.

Functional Status	N	%
Barthel index ^i^ [16]		
Total dependency	12	9.8%
Severe dependency	10	8.1%
Moderate dependency	64	52.0%
Total independency	37	30.1%
Lawton/Brody scale ^ii^ [17]		
Total dependency	39	31.7%
Severe dependency	18	14.6%
Moderate dependency	22	17.9%
Minimal dependency	21	17.1%
Total independency	23	18.7%
ECOG—Performance Status scale ^a^ [18]		
0	10	8.1%
1	40	32.5%
2	34	27.6%
3	28	22.8%
4	11	8.9%
Tinetti index ^iii^ [20]		
Increased fall risk	62	50.4%
Moderate fall risk	17	13.8%
Low fall risk	25	20.3%
Not applicable (does not walk)	19	15.4%
TUGT ^b;iv^ [21]		
<10 s	8	6.5%
10–20 s	60	48.8%
20–30 s	21	17.1%
>30 s	11	8.9%
Not evaluable	23	18.7%
Cognitive Status	N	%
MMSE ^c^ [23]		
Normal	47	38.2%
Cognitive impairment	19	15.4%
Not evaluable	57	46.3%
Nutritional Status	N	%
BMI ^d;v^ [28]		
Underweight	19	15.4%
Normal	34	27.6%
Overweight	70	56.9%
MNA ^e; vi^ [26]		
Normal	79	64.2%
Malnutritional risk	37	30.1%
Malnutrition	7	5.7%
Family and social support	N	%
Lives with family	81	65.9%
Lives alone	24	19.5%
Neighbors’ support	3	2.4%
Formal caregiver support	6	4.9%
Residential center for the elderly	9	7.3%

N: number; %: percentage; ^a^ ECOG: Eastern Cooperative Oncology Group; ^b^ TUGT: time up and go test; ^c^ MMSE: mini mental state examination; ^d^ BMI: body mass index; ^e^ MNA: mini nutritional assessment. Scales’ scoring ranges: (1) functional status: ^I^ Barthel index: total dependency (0–40), severe dependency (45–60), moderate dependency (65–95), total independency (100). ^ii^ Lawton/Brody scale—in the case of men, meal preparation, household chores and laundry are not scored: total dependency (men 0; women 0–1), severe dependency (men 1; women 2–3), moderate dependency (men 2–3; women 4–5), minimal dependency (men 4; women 6–7), total independency (men 5; women 8). ^iii^ Tinetti index: elevated fall risk (0–19), moderated fall risk (20–23), low fall risk (24–28). ^iv^ TUGT: 10 s (normal/without frailty), 10–20 s (good mobility), 20–30 s (should use an assistive device), >30 s (elevated fall risk, dependent). (2) Cognitive status: MMSE uses the patient’s educational level to score them. We considered only two categories: “normal” and “cognitive impairment”. A positive test (cognitive impairment) is indicated by a score of ≤15 points for individuals with no formal education, ≤22 points for individuals with at least 11 years of schooling and ≤27 points for those with ≥12 years of schooling. Patients scoring above these cutoffs were classified as “normal”. (3) Nutritional status: ^v^ BMI (according to Lipschitz, 1994): underweight (BMI ≤ 22), normal (BMI 23–26), overweight (BMI ≥ 27). ^vi^ MNA: normal (12–14), malnutrition risk (8–11), malnutrition (0–7).

**Table 2:** Geriatric syndromes.

Geriatric Syndromes	N	%
Polypharmacy	103	82.9%
Visual impairment	86	69.9%
Urinary incontinence	68	55.3%
Hearing loss	60	48.8%
Falls	25	20.3%
Previous fractures	25	20.3%
Weight loss	23	18.7%
Anorexia	22	17.9%
Constipation	20	16.3%
Fecal incontinence	20	16.3%
Cognitive impairment	20	16.3%
Swallowing difficulties	14	11.4%
Pressure ulcers	2	1.6%

N: number; %: percentage.

## Data Availability

The datasets presented in this article are not immediately available because the data are part of an ongoing study.
